# Concentration of Minerals in Nectar Honeys from Direct Sale and Retail in Poland

**DOI:** 10.1007/s12011-018-1315-0

**Published:** 2018-04-04

**Authors:** Monika Kędzierska-Matysek, Mariusz Florek, Anna Wolanciuk, Joanna Barłowska, Zygmunt Litwińczuk

**Affiliations:** 1 Department of Commodity Science and Processing of Animal Raw Materials, University of Life Sciences in Lublin, Akademicka 13, 20-950 Lublin, Poland; 2 Institute of Animal Breeding and Biodiversity Conservation, University of Life Sciences in Lublin, Akademicka 13, 20-950 Lublin, Poland

**Keywords:** Nectar honey, Minerals, Nutrients, Direct sale, Retail

## Abstract

The aim of the study was to compare the content of selected minerals in different nectar honeys (acacia, buckwheat, raspberry, linden, rapeseed, and multifloral) available on the Polish market. The degree to which the demand for eight minerals (K, Na, Mg, Ca, Zn, Fe, Mn, Cu) by adults is met by a portion of 100 g of honey was estimated as well. The material consisted of 34 artisanal honeys from direct sale and 34 samples purchased from retail stores. The artisanal honeys contained significantly more K, Mg, and Mn, but significantly less Na and Fe than the honeys purchased from the retail stores. The raspberry honey contained significantly the most K and Ca (1104.7 and 68.8 mg kg^−1^), the multifloral honey contained the most Ca and Mg (68.5 and 48.0 mg kg^−1^), and the buckwheat honey contained the most Zn and Mn (3.97 and 4.96 mg kg^−1^). The highest content of Na was shown in buckwheat and linden honeys (79.1 and 80.0 mg kg^−1^). Consumption of 100 g of honey from direct sale satisfied from 2.5 to 4.5% of the recommended intakes for K and from 10.4 to 17.3% for Mn, while the same portion of honey from retail satisfied from 1.6 to 4.8% for Fe, and from 2.3 to 6.1% for Zn and Cu. The buckwheat honey met to the greatest degree the recommended dietary intakes for Mn (16.5–27.6%), followed by raspberry honey (10.0–16.7%) and multifloral honey (6.9–11.6%).

## Introduction

In the era of processed food, honey is a natural product produced by *Apis mellifera* honey bees from the nectar of plants or from secretions of living parts of plants or excretions of plant-sucking insects on the living parts of plants [1]. The supply of honey in the world differs strongly in relation to regions and countries. According to FAO [2], the quantity of global honey supply in 2013 was 0.24 kg per capita, with the lowest amount in Asia and Africa (0.16–0.17 kg) and the largest amount in Europe and Oceania (0.65–0.66 kg).

Honey contains minerals essential to the proper functioning of the human body; for example, they are components of compounds that influence metabolism, participate in water-electrolyte balance, and have a regulatory effect [3]. The content of minerals in honey is significantly influenced by botanical origin [4] and ranged from 0.04 to 0.2% in floral honey [5], while that in honeydew honey varied between 0.40 and 0.63% [6]. Both the quantity and variety of minerals present in honey depend on the level of nutrients in the plants, their availability in the soil, and soil and environmental contaminations. As a consequence, an excess or deficiency of some chemical compounds in the soil or water affects the chemical composition of plants and then that of nectar [7–9]. It is also noteworthy that different elements have various concentrations in honey even from the same botanical type and collected from the same geographical region, same locality, and same beehive but in different vegetation season [10]. Moreover, an alteration relating to mineral contents may be caused by incorrect processing and conservation of honey [11]. For instance, by reason of the acidic nature of honey, the release of Al and Cd, as well other elements (such as Cr, Pb, and Zn), from metallic tools or containers may occur [12].

Bee honey can be a good source of major and trace elements needed by humans [13]. Minerals such as Ca, Cu, Fe, Mg, Mn, and Zn have very high bioavailability (80–90%) from honey [14]. Due to their role in the body, minerals should be consumed in appropriate quantities and proportions, since their dietary intakes above the safety limits may cause adverse or toxic effects [15, 16]. Also, essential metals ingested in excessive doses have toxicological implications for humans [17]. At the same time, synergistic or antagonist interactions between minerals must be taken into account, especially at the step of absorption, as these determine the bioavailability of each of them.

The aim of the study was to compare the content of selected minerals in six types of nectar honeys available on the Polish market considering the sales channels, i.e., direct sale in apiaries vs. retail stores. On the basis of results pertaining to the content of eight selected minerals in six nectar honeys, the extent to which the requirement for these minerals in adult consumers (females and males) is met by an edible portion of 100 g of honey was determined.

## Material and Methods

### Samples

The study material comprised a total of 68 nectar honeys collected from Lublin region. Honey samples were collected between May and July 2016. The 34 artisanal samples were acquired directly from the local beekeepers (Ds) produced by traditional procedures with guaranteed botanic origin and another 34 samples of honeys were purchased from a retail chain (Rs) in Lublin town, the capital city of Lubelskie Voivodeship (Lublin Province). The all products obtained from shops were appropriate labeled according to the general honey labeling rules [18]. The following honeys considering the plant origin were distinguished: acacia (*n* = 9, including Ds = 5 and Rs = 4), buckwheat (*n* = 13, including Ds = 6 and Rs = 7), raspberry (*n* = 5, including Ds = 3 and Rs = 2), linden (*n* = 12, including Ds = 5 and Rs = 7), rapeseed (*n* = 15, including Ds = 6 and Rs = 9), and multifloral (*n* = 14, including Ds = 9 and Rs = 5). The artisanal honeys were sold in sealed glass jars with a screw cap with a volume of 1 L. The honeys from the retail stores were packed into glass containers of a volume between 300 and 900 mL sealed with a screw cap. The purchased honeys were stored in a dark room at room temperature until analysis.

### Sample Preparation and Analysis

The concentration of macro- and microminerals in the honey samples was determined by atomic absorption spectrometry. Potassium (K), sodium (Na), calcium (Ca), magnesium (Mg), iron (Fe), and zinc (Zn) were determined with a Varian AA240FS Fast Sequential Atomic Absorption Spectrometer with an air-acetylene flame atomizer. Copper (Cu) and manganese (Mn) concentrations were determined with a Varian AA240Z spectrometer with a GTA-120 Graphite Tube Atomizer. For automatic volumetric dosing, the GTA system was equipped with a programmable sample dispenser (PSD).

The test samples were prepared and the equipment was washed according to the procedure given in PN-EN 13804; 0.5 to 0.8 g of honey (± 0.0001 g) was collected into flasks. Then, 6 mL of 65% suprapure nitric acid was added. Mineralization was performed under increased pressure in a CEM MARS 5 Xpress microwave digester (CEM Corporation, Matthews, NC, USA). For determination of K, Na, Ca, and Mg, a Schinkel correction buffer (Merck KGaA, Darmstadt, Germany) (10 g L^−1^ CsCl +100 g L^−1^ La) was added to the samples so that the final solution contained 1% of this buffer. During the analysis, the following detection limits (LOD) were taken into account: for Na, Mn, and Zn 0.01 mg kg^−1^; for K 0.04 mg kg^−1^; for Fe 0.09 mg kg^−1^; for Ca 0.22 mg kg^−1^; and for Mg 0.47 mg kg^−1^. The certified reference material NCS ZC 73014 Tea was tested together with the test samples to verify the accuracy of the method. The contents of the minerals were read from a calibration curve plotted from the calculated points. The analytical accuracy and precision were within 5% for all the elements. The content of macro- and microminerals in the samples was expressed in milligrams per kilogram wet mass.

### Assessment of Mineral Concentration in Relation to Nutrient Requirements

Due to the low concentration of certain nutrients, the consumption of sufficiently large amounts of honey (70–95 g per day) by adults is recommended to obtain the nutrition and health benefits [13]. On the basis of the levels determined in the present study for macro- (K, Na, Mg, Ca) and microminerals (Fe, Zn, Mn, Cu), the percentages of the recommended intakes for adult consumers were calculated for 100 g of honey. Due to the lack of common requirements the daily allowance (RDA), population reference intake (PRI), recommended nutrient intakes (RNI), daily reference intakes (DRI), and adequate intake (AI) were used for that purpose, which were developed by international and national institutions, i.e., EU [18], EFSA [19], WHO/FAO [16], and NFNI [20]. However, the WHO/FAO guidelines do not specify the recommended nutrient intakes for K, Na, Mn, and Cu.

### Statistical Analysis

Statistical analysis of the data was performed using Statistica 13 [21]. Due to the size and nature of the data, the Nonparametric Statistics package was used. The influence of the honey type (acacia, buckwheat, linden, raspberry, rapeseed, or multifloral) on the content of macro- and microminerals was verified by the Kruskal-Wallis test (comparison of many independent groups). The content of macro- and microminerals in the honey, as well within particular types, in relation to the way of sale (direct sale vs. retail) was compared by the Kolmogorov-Smirnov test (comparison of two independent groups). The tables present the mean, median, standard error of mean, and minimum and maximum values for each dependent variable. The relationship between the content of individual macro- and microminerals in the domestic flower honeys was determined by calculating Spearman’s rank correlation coefficients (*r*_S_). The correlations between the minerals were further verified by principal component analysis (PCA).

## Results and Discussion

The content of macro- and microminerals differed significantly depending on both the kind of sale (Table 1) and botanic origin (Table 2) of the honey. The observed variability of the element concentration in the honey samples was obvious. The most abundant element was K, followed by Ca, Na, and Mg. The average content of Fe, Zn, Mn, and Cu varied between 0.47 and 4.77 mg kg^−1^. Moreover, the range of variation of each mineral was usually wide. Artisanal honeys purchased directly from apiaries contained significantly more K and Mg (*P* ≤ 0.001) and Mn (*P* ≤ 0.025) and significantly less Na (*P* ≤ 0.001) and Fe (*P* ≤ 0.005) than the packaged honey purchased from the retail chain. On one hand, it should be emphasized that the honey from apiaries contained 3 times more Mn, 2.5 times more K, and 1.5 times more Mg. On the other hand, the honey from the retail chain contained more (but insignificantly) Zn and Cu and less Ca than the artisanal honeys. Considering a variability of mineral concentration it is worth noting that the mean practically coincided with the median for K, Na, Ca, Mg, Fe, and Ca in honeys from direct sale. Similar relations were noticed only for Ca and Cu in honeys from retail.

**Table 1:** Content of macro- and microminerals (in the milligrams per kilogram of honey) in nectar honeys from direct sale and retail stores

Minerals	Direct sale	Retail stores	SEM	*P* value (Kolmogorov-Smirnov test)
	*n* = 34	*n* = 34	*n* = 68	
K				
Mean	892.4*	344.0*	61.6	≤ 0.001
Median	857.4	250.3		
Range	142.4–1915.9	56.9–1044.1		
Na				
Mean	52.0*	64.0*	7.7	≤ 0.001
Median	48.3	22.0		
Range	39.7–111.7	5.9–332.1		
Ca				
Mean	61.0	55.7	2.2	> 0.05
Median	57.3	50.9		
Range	30.6–121.7	26.6–93.8		
Mg				
Mean	35.9*	22.6*	3.6	≤ 0.001
Median	31.4	14.4		
Range	9.2–74.9	4.4–239.2		
Fe				
Mean	2.17*	4.77*	0.42	≤ 0.005
Median	2.11	3.28		
Range	1.23–3.82	0.21–17.40		
Zn				
Mean	1.41	2.99	0.30	> 0.05
Median	1.08	2.00		
Range	0.03–7.56	0.07–12.45		
Mn				
Mean	3.12*	1.12*	0.34	≤ 0.025
Median	1.64	0.58		
Range	0.05–11.56	0.04–7.86		
Cu				
Mean	0.47	0.54	0.03	> 0.05
Median	0.45	0.58		
Range	0.14–0.80	0.05–1.38		

Means denoted by asterisk in rows differ significantly according to *P* value

*SEM* standard error of mean

**Table 2:** Content of macro- and microminerals (in the milligrams per kilogram of honey) in different nectar honeys

Minerals	Acacia	Buckwheat	Linden	Raspberry	Rapeseed	Multifloral	SEM	*P* value (Kruskal-Wallis test)
	*n* = 9	*n* = 13	*n* = 12	*n* = 5	*n* = 15	*n* = 14	*n* = 68	
K								
Mean	587.2^ab^	413.6^ab^	925.2^b^	1104.7^b^	265.2^a^	769.3^ab^	61.64	≤ 0.05
Median	376.2	337.2	684.3	1522.4	243.9	799.3		
Na								
Mean	53.8	79.1	80.0	48.1	31.3	54.4	7.69	> 0.05
Median	46.9	46.8	45.6	52.1	40.6	47.6		
Ca								
Mean	52.6^AB^	53.9^AB^	63.1^AB^	68.8^B^	48.9^A^	68.5^B^	2.20	≤ 0.01
Median	47.1	46.9	61.4	67.4	49.6	66.7		
Mg								
Mean	24.0^ab^	18.2^a^	28.1^ab^	47.6^ab^	19.2^ab^	48.0^b^	3.65	≤ 0.05
Median	22.4	13.4	27.9	44.9	18.3	33.8		
Fe								
Mean	2.89	4.51	3.85	2.20	3.06	3.44	0.42	> 0.05
Median	2.09	2.05	2.14	2.49	2.00	2.85		
Zn								
Mean	1.90^AB^	3.97^B^	1.33^AB^	1.24^AB^	1.07^A^	3.05^B^	0.30	≤ 0.01
Median	1.00	2.19	1.22	1.14	0.46	2.29		
Mn								
Mean	1.09^ab^	4.96^b^	1.71^ab^	3.00^b^	0.34^a^	2.08^ab^	0.34	≤ 0.05
Median	1.17	2.64	0.81	3.06	0.19	1.30		
Cu								
Mean	0.39^ab^	0.63^ab^	0.45^ab^	0.35^a^	0.63^b^	0.42^ab^	0.03	≤ 0.05
Median	0.40	0.52	0.46	0.35	0.65	0.41		

Means denoted by different letters in rows differ significantly: ^a^^,^^b^ *P* ≤ 0.05; ^A^^,^^B^ *P* ≤ 0.01

*SEM* standard error of mean

Stecka et al. [14] reported similar results to those obtained in the present study for content of Ca, Mg, Fe, Zn, and Cu (50.9, 29.2, 3.94, 3.68, and 0.55 mg kg^−1^, respectively) in commercially available Polish honey, but the content of Mn was almost three times higher (3.14 mg kg^−1^). Kek et al. [22], in an analysis of two samples of commercial honey, showed a significant difference in total mineral content (812.6 and 2357.9 mg kg^−1^). Kanoniuk et al. [23] found no influence of production environment (urban vs. non-urban) on Ca, Mg, and Fe content in flower honey from apiaries. Irrespective of the environment, the average content of Ca and Mg in honey was similar to our results; only Fe content was several times higher (36 and 22 mg kg^−1^). Formicki et al. [24] studied the content of minerals in honey from apiaries from various locations in the Małopolska Voivodship in southern Poland and found that the concentrations of individual minerals were highly varied. The content of Fe ranged from 8 to 24 mg kg^−1^, that of Mg from 42 to 86 mg kg^−1^, and that of Zn from 1.66 to 5.97 mg kg^−1^.

The results for content of minerals in honey according to the botanical origin are presented in Table 2. The significantly lowest content of K and Mn (265.2 and 0.34 mg kg^−1^, *P* ≤ 0.05), Ca and Zn (48.9 and 1.07 mg kg^−1^, *P* ≤ 0.01), and Na (31.3 mg kg^−1^, *P* > 0.05) was found in rapeseed honey, that of Cu (0.35 mg kg^−1^, *P* ≤ 0.05) and Fe (2.20 mg kg^−1^, *P* > 0.05) in raspberry honey, and that of Mg (18.2 mg kg^−1^, *P* ≤ 0.05) in buckwheat honey. Concomitantly, the buckwheat honey contained the most Zn (3.97 mg kg^−1^, *P* ≤ 0.01), Mn (4.96 mg kg^−1^, *P* ≤ 0.05), and Fe (4.51 mg kg^−1^, *P* > 0.05). The raspberry honey contained significantly (*P* ≤ 0.05) the most K and Ca (1104.7 and 68.8 mg kg^−1^), the multifloral honey contained the most Ca and Mg (68.5 mg kg^−1^, *P* ≤ 0.01 and 48.0 mg kg^−1^, *P* ≤ 0.05), and the buckwheat and linden honey had the most Na (79.1 and 80.0 mg kg^−1^, *P* > 0.05).

The results obtained are found to be consistent with findings reported by Dżugan et al. [25] for nectar honeys collected directly from apiaries localized in South–Eastern Poland in different parts of the Province of Podkarpacie. Among the microelements, the highest content of zinc was found in buckwheat honeys (2.90 mg kg^−1^), while the lowest one was in oilseed rape honeys (0.53 mg kg^−1^). Dżugan et al. [25] also found significant differences (*P* < 0.05) in the amount of manganese between buckwheat honey (7.82 mg kg^−1^) and oilseed rape honeys (0.49 mg kg^−1^), which was in agreement with present results.

The data of this study has shown that taking into consideration a variability of mineral content, the mean and median values for all elements (except K) were very similar only in raspberry honey. In acacia, linden, rapeseed, and multifloral honeys, such coincidences were found only in a few cases, whereas the buckwheat honey showed the highest variation of mineral concentrations.

When comparing the mineral concentrations of honey taking into account the way of sale within botanical types, a mere few significant differences were stated. On one hand, similar concentration of all the analyzed minerals irrespective of selling place was reported for acacia and raspberry honeys. On the other hand, rapeseed honey from direct sale in comparison to products from retail shops shown a higher content of K (373.0 vs. 193.4 mg kg^−1^, *P* ≤ 0.025), Na (47.5 vs. 20.5 mg kg^−1^, *P* ≤ 0.05), and Mg (25.6 vs. 15.0 mg kg^−1^, *P* ≤ 0.01). Similarly, higher contents of K and Mn (1437.5 vs. 559.3 mg kg^−1^ and 3.22 vs. 0.64 mg kg^−1^, *P* ≤ 0.05) were found in linden honeys from apiaries than from retail shops. The buckwheat honey from direct sale in comparison to products from retail shops contained significantly more K (516.8 vs. 313.6 mg kg^−1^, *P* ≤ 0.05) and Mg (26.5 vs. 12.3 mg kg^−1^, *P* ≤ 0.025) but less Cu (0.41 vs. 0.83 mg kg^−1^, *P* ≤ 0.01). Significantly (*P* ≤ 0.05) higher content of Zn was found in multifloral honey from retail shops compared to those from apiaries (5.6 vs. 1.5 mg kg^−1^, *P* ≤ 0.05).

Sergiel and Pohl [26] analyzed the content of minerals in different nectar honeys from Podlasie and found the lowest content of Ca, Cu, Fe, Mg, Mn, and Zn in acacia honey. Chudzińska and Baralkiewicz [27] reported higher content of K, Zn, and Cu in buckwheat honeys than in rapeseed honeys. Higher Zn concentration than that in present study was demonstrated by Roman et al. [28] in multifloral honey (3.58 mg kg^−1^) and by Przybyłowski and Wilczyńska [29] in rapeseed, linden, and buckwheat honeys (4.17, 4.33, and 6.66 mg kg^−1^, respectively) from the Pomeranian Voivodeship. Madejczyk and Baralkiewicz [30] reported substantial differences in the concentrations of K, Na, Mg, Ca, Fe, Zn, Cu, and Mn between rapeseed and forest honey.

Both the plant origin and country are significant factors determining the mineral composition of honey. Ördög et al. [31] reported similar content of K (320.06 mg kg^−1^), Mg (17.43 mg kg^−1^), Zn (1.73 mg kg^−1^), and Mn (0.23 mg kg^−1^) in Hungarian rapeseed honey, while the content of Fe (6.18 mg kg^−1^) was twice as high and that of Cu (2.02 mg kg^−1^) was three times as high as in the Polish rapeseed honey tested in the present study. Nayik and Nanda [32] reported that acacia honey from India contained 0.12 mg kg^−1^ Cu, 0.95 mg kg^−1^ Mn, 1.42 mg kg^−1^ Fe, and 0.06 mg kg^−1^ Zn, i.e., less than the concentrations obtained for this variety in the present study. Terrab et al. [33] revealed that the concentrations of Ca and Na (185.3 and 388.7 mg kg^−1^) in Spanish thyme honey were several times higher than the levels noted in Polish nectar honeys. Bilandžić et al. [34] found the significant influence of botanical origin in six different honey types in Croatia for the five essential elements (K, Ca, Mg, Fe, and Zn). Compared to present results, the linden honey from Croatia contained similar amounts of Mg and Fe (25.5 and 4.02 mg kg^−1^), considerably higher levels of K, Ca, and Zn. However, Cu concentration was almost 46 times higher than those found in the present study. To sum up, differences observed between present results and those found in the literature relating to the concentrations of the elements in the nectar honeys may also result from different methods of sample mineralization (digestion or ashing) and alternative techniques for determination of minerals.

In most cases, the positive Spearman correlation coefficients (with varying significance levels) were found between the content of individual macro- and microminerals in the honeys (Table 3). The highest positive correlation coefficients were found for K and Mg (*r*_S_ = 0.749, *P* ≤ 0.001), K and Mn (*r*_S_ = 0.630, *P* ≤ 0.001), Ca and Mg (*r*_S_ = 0.452, *P* ≤ 0.001), Mn and Mg (*r*_S_ = 0.384, *P* ≤ 0.01), and K and Ca (*r*_S_ = 0.328, *P* ≤ 0.01). Significant (*P* ≤ 0.01) and negative correlations were found only for Cu and Na (*r*_S_ = − 0.333) and for Cu and Ca (*r*_S_ = − 0.336). Kacaniová et al. [35] in samples of blossom, blend, and honeydew honeys obtained in Slovakia found significant correlations between Ca and Mg, and Ca and Zn similar as in the present study, as well between Cu and Zn, and Cu and Mg. A strong positive correlation between K and Cu (*r* = 0.80), while a less strong correlation between Mg and Cu (*r* = 0.71), was also reported by Dżugan et al. [25] for different nectar (e.g., multifloral, oilseed rape, linden, buckwheat) and honeydew honeys from apiaries localized in Podkarpacie Province (South–Eastern Poland). Moreover, the cited authors concluded that the specific chemical composition and properties of soil influenced the honey mineral content more than the botanical origin.

**Table 3:** Spearman’s rank correlation coefficients (*r*_S_) between the concentrations of minerals in nectar honeys

Mineral	K	Na	Ca	Mg	Fe	Zn	Mn	Cu
K	–	0.096	0.328**	0.749***	0.016	− 0.109	0.630***	0.013
Na	0.096	–	0.181	0.270*	0.178	0.183	0.257*	− 0.333**
Ca	0.328**	0.181	–	0.452***	0.217	0.263*	0.224	− 0.336**
Mg	0.749***	0.270*	0.452***	–	0.057	− 0.156	0.384**	− 0.153
Fe	0.016	0.178	0.217	0.057	–	0.159	0.140	0.181
Zn	− 0.109	0.183	0.263*	− 0.156	0.159	–	0.228	− 0.111
Mn	0.630***	0.257*	0.224	0.384**	0.140	0.228	–	0.015
Cu	0.013	− 0.333**	− 0.336**	− 0.153	0.181	− 0.111	0.015	–

*P ≤ 0.05; **P ≤ 0.01; ***P ≤ 0.001

**Fig. 1:**
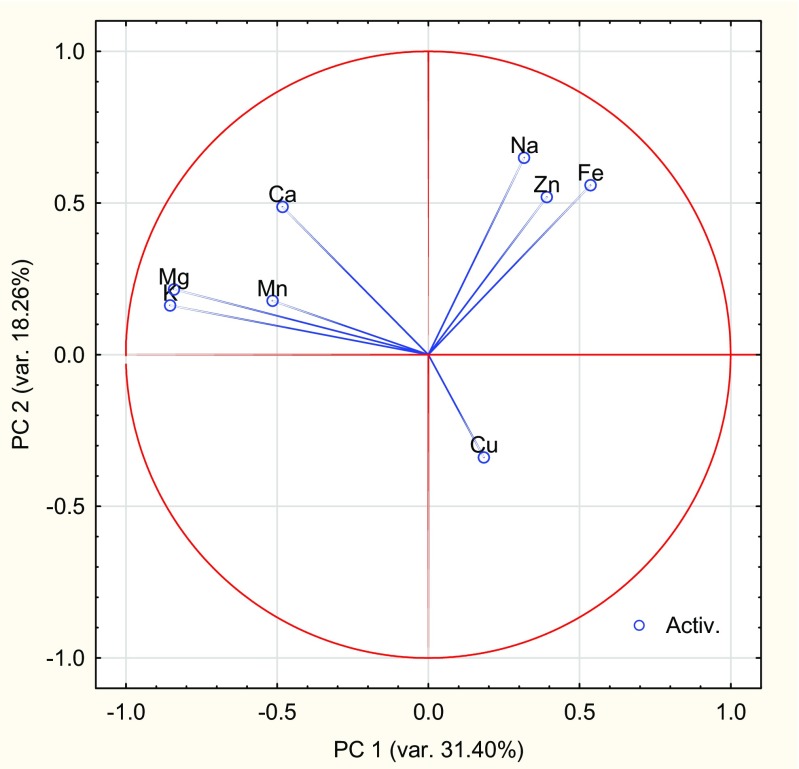
Correlations (and circle of correlations) of the variables with components 1 and 2

The differences among different honey types on grounds of the kind of sale and botanical origin were highlighted by the PCA analysis. In the present study, 8 variables and 68 cases were included in the PCA procedure. Taking into account the Kaiser criterion (eigenvalue > 1) and the screen plot, three main components were selected, which explain 66% of the total variance (Table 4). Figure 1 visualizes the projection of variables onto a two-factor plane (PC1 × PC2), explaining 49.66% of the total variance. Due to distinctive loadings (the coordinates of the points on the graph) and the length of the directional vectors connecting the points representing the variables with the origin of the coordinate system, the three groups of minerals can be distinguish. Table 5 shows the correlation coefficients between the factors and variables obtained from the correlation matrix. The K and Mg variables distributed in the negative area of the first component (PC1), explaining 31.4% of the total variance, have high negative coefficients (− 0.854 and − 0.842). The Na and Fe have high coefficients (0.650 and 0.559) in the positive area for the second component (PC2). Only the Cu variable had the highest negative coefficient (− 0.768) with the third component (PC3) and was negatively correlated with Ca.

**Table 4:** Eigenvalues and the proportion of variation (%) explained by the principal components

Component	Eigenvalue	Proportion	Cumulative
1	2.51	31.40	31.40
2	1.46	18.26	49.66
3	1.31	16.37	66.02
4	0.96	12.05	78.07
5	0.69	8.68	86.75
6	0.50	6.23	92.99
7	0.36	4.54	97.53
8	0.20	2.47	100.00

**Table 5:** Correlations between the principal components and the original variables

Variable	Principal component		
	1	2	3
K	− 0.854	0.162	− 0.294
Mg	− 0.842	0.215	− 0.085
Mn	− 0.516	0.178	− 0.493
Ca	− 0.483	0.488	0.412
Na	0.317	0.650	− 0.225
Fe	0.536	0.559	− 0.388
Zn	0.392	0.520	0.107
Cu	0.184	− 0.338	− 0.768

**Fig. 2:**
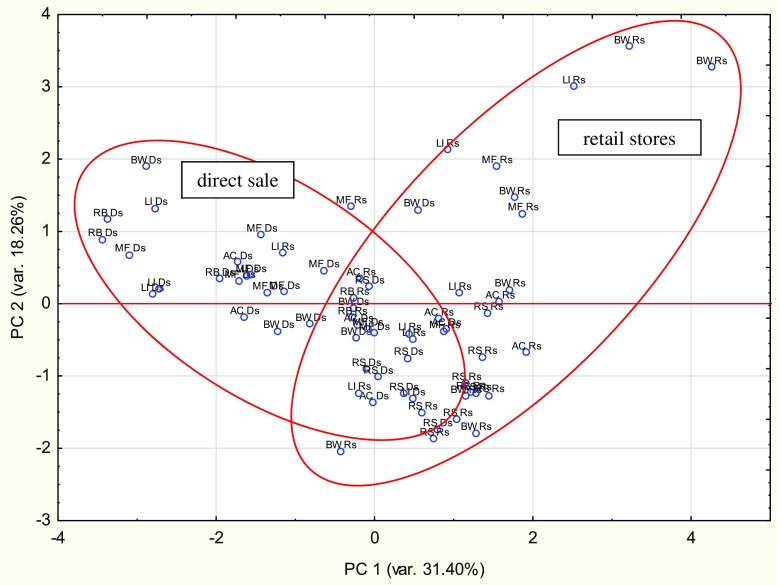
Plot of 68 honey samples with respect to their first two PCs (*AC* acacia, *BW* buckwheat, *RB* raspberry, *LI* linden, *RS* rapeseed, *MF* multifloral, *Ds* direct sale, *Rs* retail stores)

Figure 2 shows the projection of cases depending on the selling place of the honey (Ds—direct sale, Rs—retail stores) and botanical origin (AC—acacia, BW—buckwheat, RB—raspberry, LI—linden, RS—rapeseed, MF—multifloral) in the coordinate system defined by the main factors 1 and 2. While the two groups of honey are not fully separated from one another, they are grouped together depending on the place where the products were sold. The different concentrations of minerals in honey from compared vendors could be related to disparate equipment and processing operations after collecting of product in order to prepare it for distribution. Honey bought in stores was characterized by positive values along the first axis and both negative and positive values along the second axis. Most of the artisanal honeys purchased directly from apiaries had positive values for the first and negative values for the second axis and positive values for the second and negative values for the first axis. In sum, the data presented in Figs. 1 and 2 confirm the results given in Table 1. Honey obtained directly from the apiary contained significantly more K, Mg, and Mn, while the packaged honey from shops contained significantly more Na and Fe. In addition, there was a negative correlation between Cu and Ca. Pisani et al. [12], in their analysis of the content of microminerals and trace elements in various types of Italian honeys, confirmed that their botanical origin significantly influenced their chemical composition, particularly in the case of Ca, Na, and Mn. Furthermore, PCA analysis indicates correlations between the concentration of minerals and the type of honey.

The recommended intakes (according to different references) for dietary macro- and microminerals, as well the percentages of requirements met by 100 g of nectar honeys in different groups of adults, are shown in Tables 6 and 7, respectively. It must be pointed out that natural contribution of the portion of 100 g of studied honeys in terms of essential elements for an adult varied greatly in accordance with the kind of sale and botanic origin of honey. On one hand, consumption of artisanal honeys can fulfill requirements for K (from 2.5 to 4.5% of the recommended intakes) and Mn (from 10.4 to 17.3%) in higher percentage than intake of honey purchased in retail shops. On the other hand, 100 g of honeys from the latter source satisfies the nutritional requirements for Fe from 1.6 to 4.8% and for Zn and Cu from 2.3 to 6.1%. Irrespective of kind of sale, honey met only 0.2–1.6% of the daily standard for Na, Mg, and Ca for adults. In general, present results coincide with the findings obtained by Altundag et al. [36] for nutritional value of Turkish local and commercial honeys. The content of essential elements in honey was very low, i.e., for Cu from 0.45 to 2.15 mg kg^−1^, for Zn from 0.80 to 12.03 mg kg^−1^, for Mn from 0.13 to 15.02 mg kg^−1^, and for Fe from 3.87 to 16.76 mg kg^−1^. For that reason, the average daily consumption of honey (about 20 g) contained only 1% of the RDA values for these elements. For that reason, the average daily consumption of honey (about 20 g) contained only 1% of the RDA values for these elements. In present study, 100 g of honey was used for calculation, and consequently, such portion covered the recommended intakes for microminerals five times more, respectively.

**Table 6:** Recommended intakes for dietary macrominerals and percentage of requirements for these elements met by 100 g of nectar honeys in different groups of adults

Mineral	Requirements and references	Group	Unit (mg/day)	Kind of sale		Botanic origin of honey					
				Direct sale	Retail	Acacia	Buckwheat	Linden	Raspberry	Rapeseed	Multifloral
K	AI [19]	Females > 18	3500	2.5	1.0	1.7	1.2	2.6	3.2	0.8	2.2
		Males > 18	3500	2.5	1.0	1.7	1.2	2.6	3.2	0.8	2.2
	RNI [16]	Females 19–65	GA	–	–	–	–	–	–	–	–
		Males 19–65	GA	–	–	–	–	–	–	–	–
	DRI [18]	Adults	2000	4.5	1.7	2.9	2.1	4.6	5.5	1.3	3.8
	AI [20]	Females 19–65	3500	2.6	1.0	1.7	1.2	2.6	3.2	0.8	2.2
		Males 19–65	3500	2.6	1.0	1.7	1.2	2.6	3.2	0.8	2.2
Na	AI [19]	Females > 18	2400	0.2	0.3	0.2	0.3	0.3	0.2	0.1	0.2
		Males > 18	2400	0.2	0.3	0.2	0.3	0.3	0.2	0.1	0.2
	RNI [16]	Females 19–65	GA	–	–	–	–	–	–	–	–
		Males 19–65	GA	–	–	–	–	–	–	–	–
	DRI [18]	Adults	2400	0.2	0.3	0.2	0.3	0.3	0.2	0.1	0.2
	AI [20]	Females 19–65	1500	0.3	0.4	0.4	0.5	0.5	0.3	0.2	0.4
		Males 19–65	1500	0.3	0.4	0.4	0.5	0.5	0.3	0.2	0.4
Mg	AI [19]	Females > 18	300	1.2	0.8	0.8	0.6	0.9	1.6	0.6	1.6
		Males > 18	350	1.0	0.6	0.7	0.5	0.8	1.4	0.5	1.4
	RNI [16]	Females 19–65	220	1.6	1.0	1.1	0.8	1.3	2.2	0.9	2.2
		Males 19–65	260	1.4	0.9	0.9	0.7	1.1	1.8	0.7	1.8
	DRI [18]	Adults	375	1.0	0.6	0.6	0.5	0.7	1.3	0.5	1.3
	RDA [20]	Females 19–65	320	1.1	0.7	0.8	0.6	0.9	1.5	0.6	1.5
		Males 19–65	420	0.9	0.5	0.6	0.4	0.7	1.1	0.5	1.1
Ca	PRI [19]	Females > 18	1000	0.6	0.6	0.5	0.5	0.6	0.7	0.5	0.7
		Males > 18	1000	0.6	0.6	0.5	0.5	0.6	0.7	0.5	0.7
	RNI [16]	Females 19–65	1000	0.6	0.6	0.5	0.5	0.6	0.7	0.5	0.7
		Males 19–65	1000	0.6	0.6	0.5	0.5	0.6	0.7	0.5	0.7
	DRI [18]	Adults	800	0.8	0.7	0.7	0.7	0.8	0.9	0.6	0.9
	RDA [20]	Females 19–65	1100	0.6	0.5	0.5	0.5	0.6	0.6	0.4	0.6
		Males 19–65	1000	0.6	0.6	0.5	0.5	0.6	0.7	0.5	0.7

Salt = sodium × 2.5

*PRI* population reference intake, *AI* adequate intake, *RNI* recommended nutrient intakes, *DRI* daily reference intakes, *RDA* recommended dietary allowances, *GA* the guide’s absence

**Table 7:** Recommended intakes for dietary microminerals and percentage of requirements for these elements met by 100 g of nectar honeys in different groups of adults

Mineral	Requirements and references	Group	Units (mg/day)	Kind of sale		Botanic origin of honey					
				Direct sale	Retail	Acacia	Buckwheat	Linden	Raspberry	Rapeseed	Multifloral
Fe	PRI [19]	Females > 18	11	2.0	4.3	2.6	4.1	3.5	2.0	2.8	3.1
		Males > 18	11	2.0	4.3	2.6	4.1	3.5	2.0	2.8	3.1
	RNI [16]	Females 19–65	13.7	1.6	3.5	0.2	3.3	2.8	1.6	2.2	2.5
		Males 19–65	29.4	0.7	1.6	1.0	1.5	1.3	0.7	1.0	1.2
	DRI [18]	Adults	14	1.6	3.4	2.1	3.2	2.8	1.6	2.2	2.5
	RDA [20]	Females 19–65	18	1.2	2.7	1.6	2.5	2.2	1.2	1.7	1.9
		Males 19–65	10	2.2	4.8	2.9	4.5	3.9	2.2	3.1	3.4
Zn	PRI [19]	Females > 18	10	1.4	3.0	1.9	4.0	1.3	1.2	1.1	3.1
		Males > 18	13	1.1	2.3	1.5	3.1	1.0	1.0	0.8	2.3
	RNI [16]	Females 19–65	4.9	2.9	6.1	3.9	8.1	2.7	2.5	2.2	6.2
		Males 19–65	7.0	2.0	4.3	2.7	5.7	1.9	1.8	1.5	4.4
	DRI [18]	Adults	10	1.4	3.0	1.9	4.0	1.3	1.2	1.1	3.1
	RDA [20]	Females 19–65	8	1.8	3.7	2.4	5.0	1.7	1.6	1.3	3.8
		Males 19–65	11	1.3	2.7	1.7	3.6	1.2	1.1	1.0	2.8
Mn	AI [19]	Females > 18	3	10.4	3.7	3.6	16.5	5.7	10.0	1.1	6.9
		Males > 18	3	10.4	3.7	3.6	16.5	5.7	10.0	1.1	6.9
	RNI [16]	Females 19–65	GA	–	–	–	–	–	–	–	–
		Males 19–65	GA	–	–	–	–	–	–	–	–
	DRI [18]	Adults	2	15.6	5.6	5.5	25.0	8.6	15.0	1.7	10.4
	AI [20]	Females 19–65	1.8	17.3	6.2	6.0	27.6	9.5	16.7	1.9	11.6
		Males 19–65	2.3	13.6	4.9	4.7	21.6	7.4	13.0	1.5	9.0
Cu	AI [19]	Females > 18	1.3	3.6	4.2	3.0	4.8	3.5	2.7	4.8	3.2
		Males > 18	1.6	2.9	3.4	2.4	3.9	2.8	2.2	3.9	2.6
	RNI [16]	Females 19–65	GA	–	–	–	–	–	–	–	–
		Males 19–65	GA	–	–	–	–	–	–	–	–
	DRI [18]	Adults	1	4.7	5.4	3.9	6.3	4.5	3.5	6.3	4.2
	RDA [20]	Females 19–65	0.9	5.2	6.0	4.3	7.0	5.0	3.9	7.0	4.7
		Males 19–65	0.9	5.2	6.0	4.3	7.0	5.0	3.9	7.0	4.7

*PRI* population reference intake, *AI* adequate intake, *RNI* recommended nutrient intakes, *DRI* daily reference intakes, *RDA* recommended dietary allowances, *GA* the guide’s absence

Among the evaluated nectar honeys, the buckwheat honey met to the greatest degree the recommended dietary intakes for Mn (between 16.5 and 27.6%), followed by raspberry honey (10.0–16.7%) and multifloral honey (6.9–11.6%). In general, nectar honeys satisfied in greater extent (between 2 and 5%) the nutritional standards for Fe, Zn, and Cu, and linden and raspberry honeys for K, than for Na, Mg, and Ca (up to 1%).

## Conclusions

The present study showed that the kind of sale (direct vs. retail) affected the concentration of K, Mg, Mn, Na, and Fe in the honey. The plant origin of honey significantly influenced the content of all minerals (except Na and Fe). The raspberry honey contained the highest amounts of K, Ca, and Mg, and the buckwheat honey the highest amounts of Zn, Mn, and Fe. Concentration of Mg was positively and significantly correlated with the content of Mn, Ca, and K (0.384 ≤ *r*_S_ ≤ 0.749), and that of K with the content of Mn (*r*_S_ = 0.630) and Ca (*r*_S_ = 0.328). The Cu content was negatively and significantly correlated with Na and Ca. The PCA analysis confirmed that the concentration of minerals depended on the kind of sale and botanical origin of the honey. For nutritional purposes, it was found that buckwheat, raspberry, and multifloral honeys were moderate sources only of Mn for adult population. The present study showed that the evaluated nectar honeys satisfied to a higher degree the standards for microminerals, than macronutrients.
